# Diversity of the fecal microbiota in Chinese ponies

**DOI:** 10.3389/fvets.2023.1102186

**Published:** 2023-01-26

**Authors:** Shipeng Lv, Yanli Zhang, Zhengkai Zhang, Sihan Meng, Yabin Pu, Xuexue Liu, Lingling Liu, Yuehui Ma, Wujun Liu, Lin Jiang

**Affiliations:** ^1^College of Animal Science, Xinjiang Agricultural University, Urumqi, China; ^2^Laboratory of Animal (Poultry) Genetics Breeding and Reproduction, Ministry of Agriculture, Institute of Animal Science, Chinese Academy of Agricultural Sciences (CAAS), Beijing, China; ^3^CAAS-ILRI Joint Laboratory on Livestock and Forage Genetic Resources, Institute of Animal Science, Chinese Academy of Agricultural Sciences (CAAS), Beijing, China; ^4^Centre d'Anthropobiologie et de Génomique de Toulouse, Université Paul Sabatier, Toulouse, France

**Keywords:** equine, 16S rRNA sequencing, fecal microbiota transplantation, intestinal microorganisms, microbial community diversity

## Abstract

**Introduction:**

The gut microbiomes of equine are plentiful and intricate, which plays an important part in the growth. However, there is a relative lack of information on the microbial diversity in the pony's gut.

**Methods:**

In this article, 118 fecal samples from DeBa pony, NiQi pony and GuZh horse were studied by 16S rRNA amplicon sequencing.

**Results:**

Diversity analysis was used to determine the difference of gut microbiota composition among different breeds. Alpha diversity analysis showed that the gut microbiota of NiQi ponies were abundant and various. Beta diversity analysis showed that the microorganisms constitution of DeBa ponies was more similar to that of NiQi ponies. LDA Effect Size (LEfSe) analysis result that the microorganism biomarkers for NiQi pony at the genus level were Phascolarctobacterium, Paludibacter, and Fibrobacter; the bacterial biomarker for DeBa pony was Streptococcus and Prevotella; and the bacterial biomarkers for GuZh horses was Treponema, Treponema Mogibacterium, Adlercreutzia, and Blautia. The correlation analysis between genera with >1% abundance and horse height found that Streptococcus (*P* < 0.01), Treponema (*P* < 0.01), Coprococcus (*P* < 0.01), Prevotella (*P* < 0.01), Phascolarctobacterium (*P* < 0.01), and Mogibacterium (*P* < 0.01) were significantly associated with horses' height. The functional prediction results indicated that DeBa pony have a microbiota functional more similar to NiQi pony.

**Discussion:**

For the first time, our results announce the species composition and structure of the gut microbiota in Chinese ponies. At the same time, our results can provide theoretical reference for further understanding the healthy breeding, feeding management and disease prevention of horses.

## Introduction

Chinese ponies are mainly distributed in the mountainous areas of southwest China, which is one of the most famous ponies producing areas in the world. On the basis of providing important means of production for agricultural production, ponies have also promoted the development of rural tourism industry mainly for children's entertainment and sightseeing. With the development of society and the improvement of people's quality of life, people began to pursue a richer spiritual and cultural life. Because ponies are gentle, intelligent, and have the attributes of riding, racing and ornamentation, they have higher value, so the health of ponies is greatly concerned, especially their intestinal health. In fact, it is widely known that domestic horses were vulnerable to diseases originating in the gut, while the microbiota in the gut were prone to disturbances and malfunctions (1).

Research in recent decades has highlighted the importance of the microbiota in the normal development and physiological development and maintenance of the gut, including digestion and nutrient absorption, metabolism, tissue development, and immunity (2). The animal gastrointestinal tract is home to a large and diverse microflora, and constitutes a large and complex system (3). As an ecosystem where organisms coexist, microorganisms are considered the “second largest gene pool” of genetic information for animals (4), forms a complete organism with the host (5). Compared with the widely studied human microflora, animal microflora has received less attention (6). For herbivores, they are unable to synthesize in their own bodies the hydrolytic enzymes needed to degrade plant lignocellulose (7). In general, it is often the microorganisms in the organism that convert indigestible feed into easily absorbed compounds, thus providing nutrition to the host and effectively promoting the physiological health of the host. Thus, by better understanding the equine microbiome, we can inform interventions that will improve health and welfare, performance, value and longevity of the horse.

One study found that host genetics, diet and geography affect the structure of the gut microbial community (8). It has since been sufficient established that gut microbiota plays an importance role in nutritional absorption, vitamin synthesis, food digestion, energy harvest, and metabolism in humans and other animals (9). The horse gut microbiota is a complex ecosystem comprised of thousands of microorganisms (10, 11). Through the microbiological analysis of the feces of Mongolian and Thoroughbred horses, it was found that there were significant differences in 5 phyla and 30 genera (12). Another research found that Przewalski horse fecal microbiomes have a distinct and more diverse community of bacteria compared with the domestic equine (13). There is also study analyzed the gut microbiota of Quarter horse, Azteca, warmblood, Thoroughbreds, and Andalusian breeds and found that the family Christensenellaceae has been found in animals belonging exclusively to the Thoroughbreds breed (14). A recent investigation has revealed diversity in the microbiome of equine from different geographical situation (15). However, those researches are limited to tall horses, and there is lack of understanding of the intestinal flora of ponies.

Animal fecal samples are typically used as a substitute for intestinal microflora and have been widely used in the study of intestinal microorganisms in animals (16–19). Here, we used 16S rRNA amplicon sequencing to research the microorganism in the feces of equine from three different region, we collecte118 fecal samples from Guangxi and Shannxi provinces in China. First, we investigate the diverse in the community structure and species composition of the gut microbiota of pony and tall horses; secondly, to explore the relationship between gut microbes and body height in horses.

## Materials and methods

### Animals and sample collection

All animal work was conducted according to the guidelines for the care and use of experimental animals established by the Ministry of Agriculture of China. And all methods are reported in accordance with ARRIVE guidelines (https://arriveguidelines.org) for the reporting of animal experiments. The project was also approved by Animal Care and Use Committee (ACUC) in Institute of Animal Sciences, Chinese Academy of Agricultural Sciences (ethical permit: IAS2019-24). Our study included 118 healthy horses. There were including Debao (DeBa) ponies (*n* = 31), Ningqiang (NiQi) ponies (*n* = 47) and Guanzhong (GuZh) horses (*n* = 40) (Supplementary Table S1). All horses were house fed forage and concentrate supplement. The adult animals were selected based on the following criteria: no drugs affecting gastrointestinal microbes were used within 6 months, no reported illness within the past 6 months of the study and, no gut-related disorders recorded until the beginning of the study. All the samples are in the same growing stage. Furthermore, the animals were clinically healthy based on their parasite profiles. One hundred and eighteen fecal samples were collected from the rectum of horses using long arm gloves. Samples were aliquoted into 2 ml cryovials and immediately snap frozen in liquid nitrogen. Then take it to the laboratory and store it in −80°C refrigerator until DNA extraction.

### DNA extraction, library construction, and sequencing

Microbiome DNA was extracted with the Omega Stool DNA kit (Omega, Norcross, GA, USA) follow the appropriate instructions. The DNA concentration and purity were quantified with a Nanodrop 2000^®^ (ThermoFisher Scientific, USA) and Qubit3.0 (Life Invitrogen, USA), respectively. 1% agarose gel electrophoresis was used to examined DNA quality. According to the literature describe (20, 21), We amplified the v3-v4 (338F-806R) region of the 16S rRNA gene. Brief, the PCR components contained 5 μl of buffer (5×), 0.25 μl of Fast pfu DNA, Polymerase (5 U/μl), 2 μl (2.5 mM) of dNTPs, 1 μl (10 uM) of each Forward and Reverse primer, 1 μl of DNA Template, and 14.75 μl of ddH2O. The PCR amplification program is carried out according to the Hai-Bin Yang program (20), as follows: 98°C for 5 min, followed by 25 cycles consisting of denaturation at 98°C for 30 s, annealing at 53°C for 30 s, and extension at 72°C for 45 s, with a final extension of 5 min at 72°C (20, 21). PCR amplicons were purified with Vazyme VAHTSTM DNA Clean Beads (Vazyme, Nanjing, China) and quantified using the Quant-iT Pico Green dsDNA Assay Kit (Invitrogen, Carlsbad, CA, USA) (20, 21). After the individual quantification step, amplicons were pooled in equal amounts, and pair-end 2×250 bp sequencing was performed using the Illlumina MiSeq platform with MiSeq Reagent Kit v3 at Shanghai Personal Biotechnology Co., Ltd (Shanghai, China).

### Sequence analysis

QIIME2 (Quantitative Insights into Microbial Ecology), version 2019.04 (22) were used to process sequences. Analyze according to the guidance of the official website (https://docs.qiime2.org/2019.4/tutorials/). Removal of primers, quality control, denoising, splicing of double-terminal sequences, removal of chimera and identification of amplicon sequence variants (ASVs) were performed using DADA (23). For taxonomic classification, we selected the Greengenes database (version 13.8) using the Naïve Bayes classifier in QIIME2 (24, 25). ASVs that were identified in only a single sample or classified as non-bacterial were discarded. The sequence of each horse was randomly selected to achieve a uniform sequencing depth for fair comparison (26).

### Statistical analysis of data

Rarefaction curves were plotted for each sample to determine the abundance of communities and sequencing data of each sample (27, 28). The QIIME2 software has been used to calculate alpha diversity index and evaluated by Observed_species index and Shannon index (29). The Observed_species index is used to evaluate species of abundance, and the higher the value of the index, the more species are included in the sample. The Shannon index measure species diversity, affect the species richness and evenness in the sample microbial community. Differences in alpha diversity indices between groups were analyzed by Kruskal-Wallis Rank sum test, and P < 0.05 was considered significant (30). Beta diversity measurements, including gut microbiota trees, were calculated as described to compare species diversity between different samples (31). Bacterial taxonomic distributions of sample communities were visualized using the R package software. Bacterial biomarkers with markedly different abundance between horses and ponies were analyzed using the LEfSe (linear discriminant analysis effect size) method. LDA (Linear discriminant analysis) was performed on the identified divergent species to estimate the effect size of each divergent species abundance on the difference between groups, with LDA scores >3.5 (32). The relationship between genera and body height was statistically analyzed using Pearson correlation. *P* < 0.05 was considered statistically significant (32).

## Results

### Bacterial composition of horses fecal

Microbial genomic DNA was obtained from 118 samples, and the V3–V4 region of the 16S rRNA was sequenced. The reads of 118 samples were denoised, and a total of 6,657,888 clean reads were obtained. In total, 211, 683 ASVs were obtained by DADA2 software and were annotated 24 phylum and 368 genera. The Shannon-Wiener and grade abundance curves generated by R software are shown in Supplementary Figures S1A–F. This result indicates that the number of ASVs per sample is relatively homogeneous.

### Analysis of microbial diversity in the equine gut

**Figure 1:**
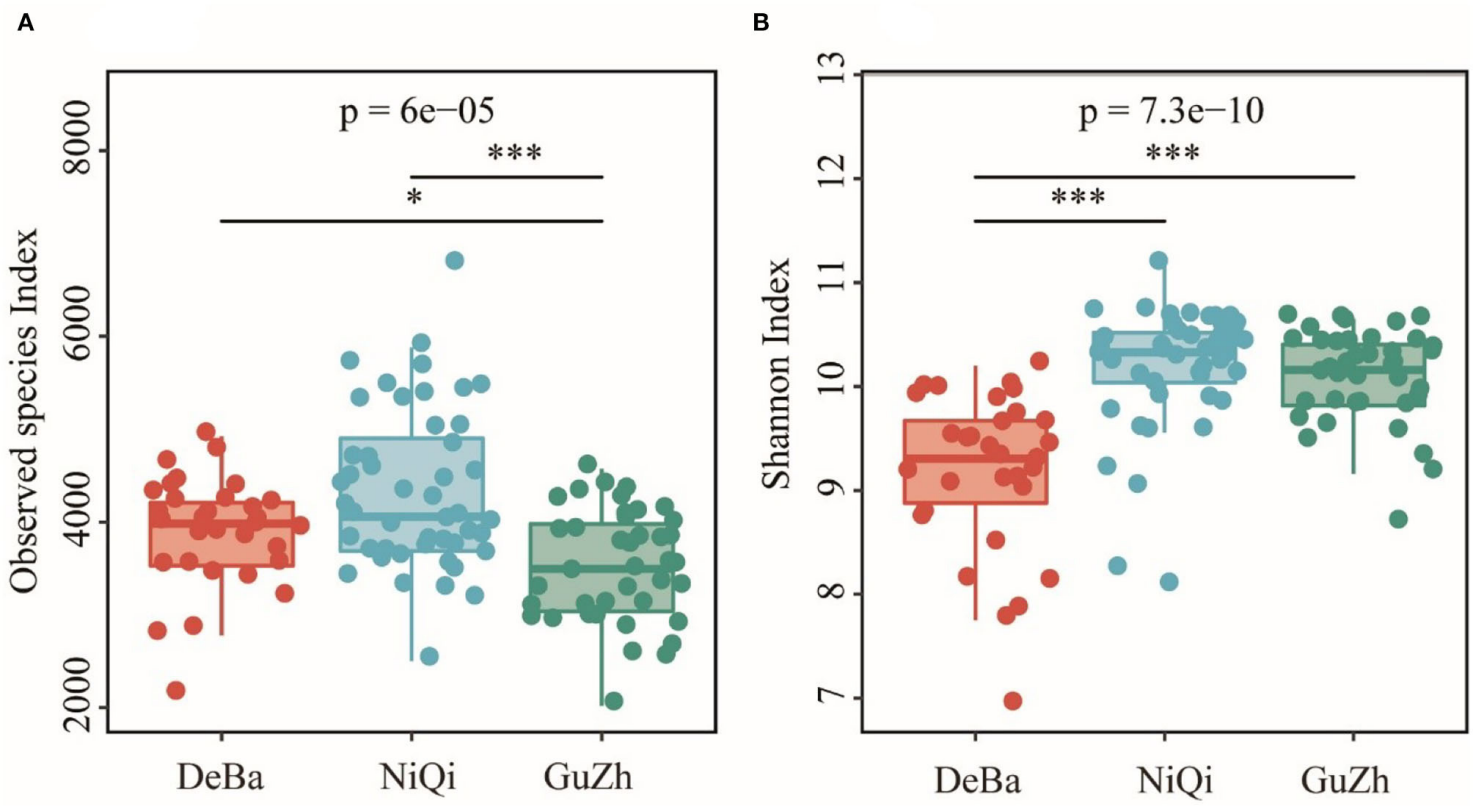
Differential analysis of the alpha diversity index. **(A)** Observed_species index. **(B)** Shannon index. Statistical method: one-way ANOVA with Tukey's *post-hoc* test. ***P < 0.001, *P < 0.05.

The Good's coverage of DeBa ponies, NiQi ponies, and GuZh horses was 0.952 7, 0.942 6, and 0.958 6, respectively, indicating that the proportion of undetected species in the sample is relatively small (Supplementary Figure S2). The Observed species index of DeBa ponies and NiQi ponies was significantly higher than those in GuZh horses (Figure 1A). The Shannon index of DeBa ponies was 9.15, which was significantly lower than that in NiQi ponies (10.16, *P* < 0.01) and GuZh horses (10.07, *P* < 0.01) (Figure 1B). The statistical analysis showed that NiQi ponies had a richer and more various gut microbiota. Interestingly, DeBa ponies had more observed species than GuZh ponies, the shannon index value of DeBa ponies was smaller than that of GuZh. The reason for this phenomenon may have something to do with the distribution of horses. DeBa ponies are distributed in the south and GuZh horses in the north (8).

**Figure 2:**
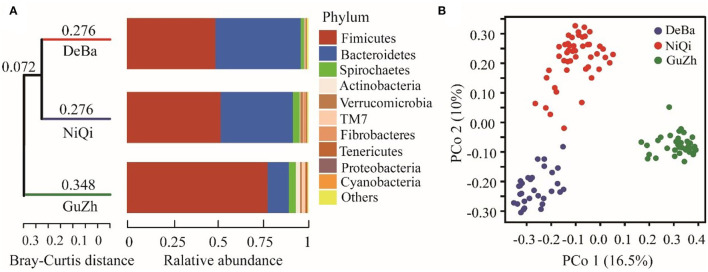
Relationship of the gut microbiota of the equines from three populations. **(A)** UPGMA cluster tree based on Bray-Curtis analysis of the structure of intestinal microorganisms in the three horses breeds. The UPGMA (unweighted pair group method with arithmetic mean) cluster tree structure is shown on the left, and the relative abundance distribution map of species at the gate level of each sample is shown on the right. **(B)** Maps representing the beta diversity based on Bray-Curtis analysis. Plots are generated base on the Bray-Curtis distance. Blue dots represent the Debao (DeBa) group, red dots represent the Ninqiang (NiQi) group and green dots represent the Guanzhong (GuZh) group.

We investigated the relationship between the 118 feces samples from three different regions using Bray–Curtis distances. The UPGMA cluster tree of intestinal microbial structure of three horse breeds was drawn. Each subfield on the tree represented one regions of gut microbiota. Even more interesting is that the gut microbiota of the DeBa pony and NiQi pony clustered together, but those of the GuZh horse located on different subfields (Figure 2A).

We used PCoA (principal coordinate analysis) to examine the gut microbiotas community structures of the three breeds equines. On the PCoA plot (Figure 2B), the bacterial communities from the DeBa pony and NiQi pony clustered tightly and were separated from those from the GuZh horse along principal coordinate axis 1 (PC1), and cluster analysis was similar, which explained the largest amount of variation (16.5%). This result indicating that the composition of intestinal microorganisms in animals of the same body size tends to be similar, which is consistent with the findings of previous studies (33).

### Horse gut microbial composition

**Figure 3:**
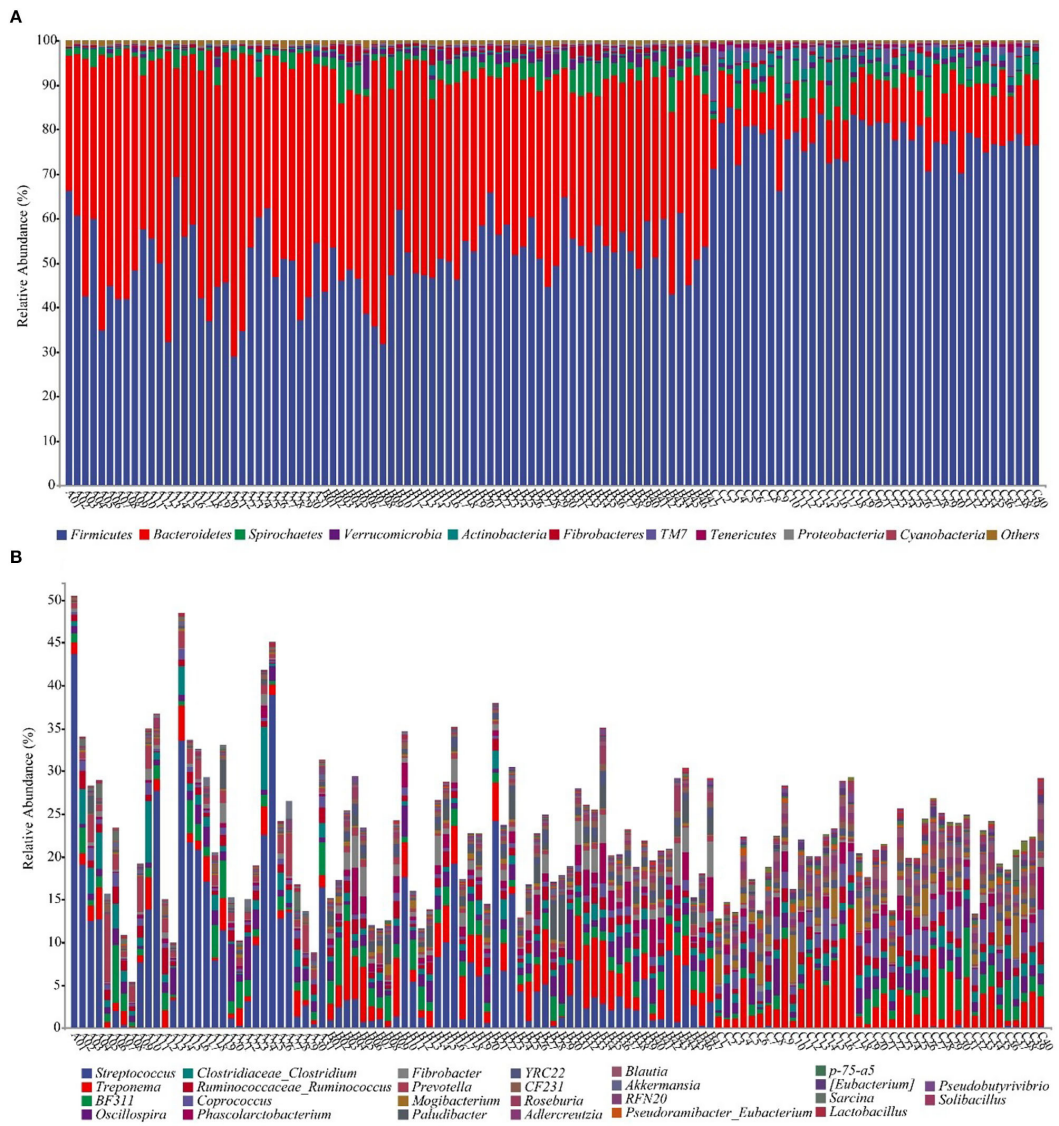
Fecal bacterial community at the phylum **(A)** and genus **(B)** levels. **(A, B)** Relative abundance of bacterial groups in the feces of 118 equines. Less than 1% abundance of the phyla was merged into others. Thirty-one samples from DeBa pony (A1–A31), 47 samples from NiQi pony (B1–B47), and 40 samples from GuZh horse (C1–C40).

**Figure 4:**
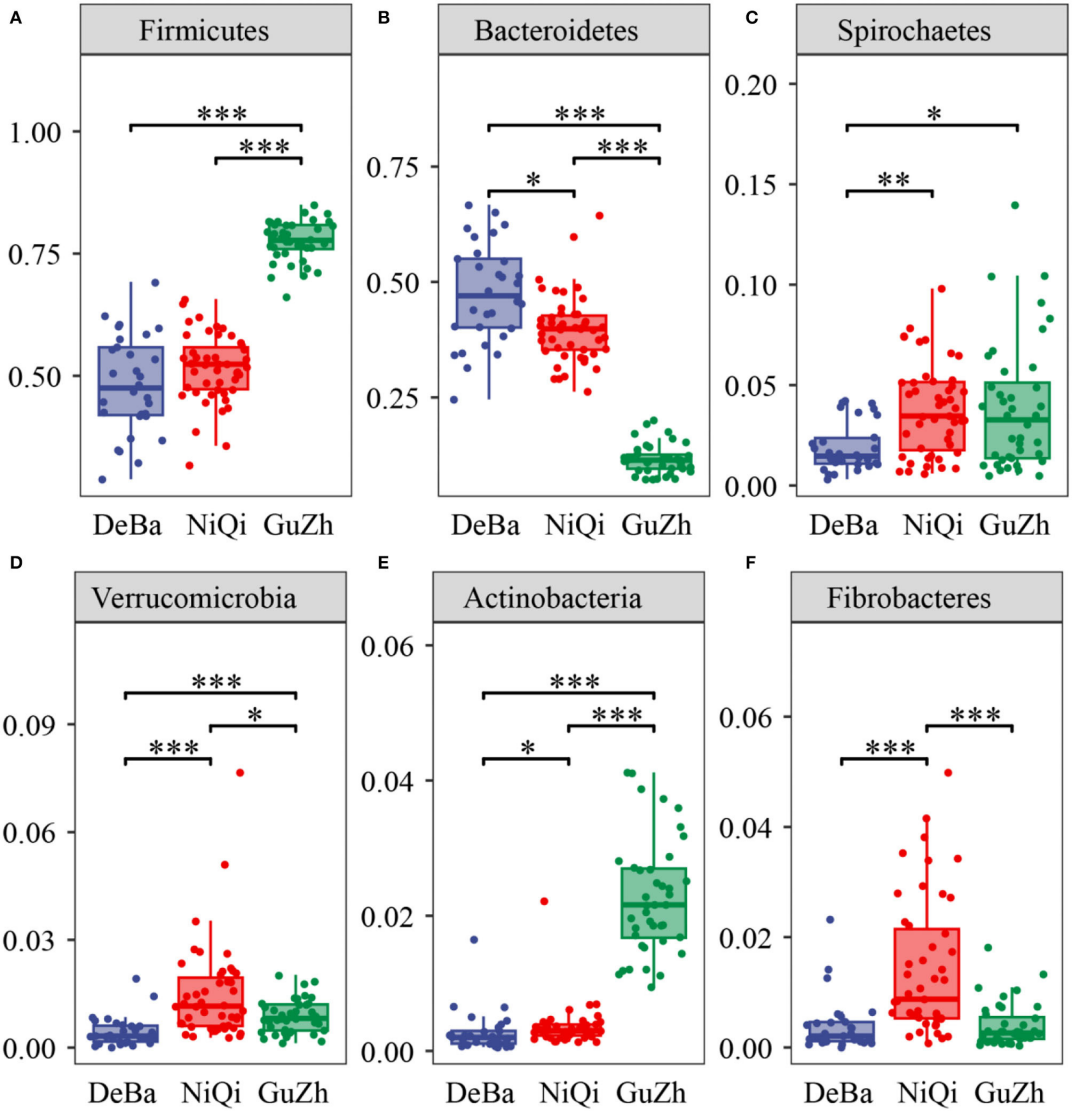
Statistical comparison of the relative abundance. **(A–F)** Comparison of dominant phyla in the DeBa, NiQi, and GuZh groups (***P < 0.001, **P < 0.01, *P < 0.05).

**Figure 5:**
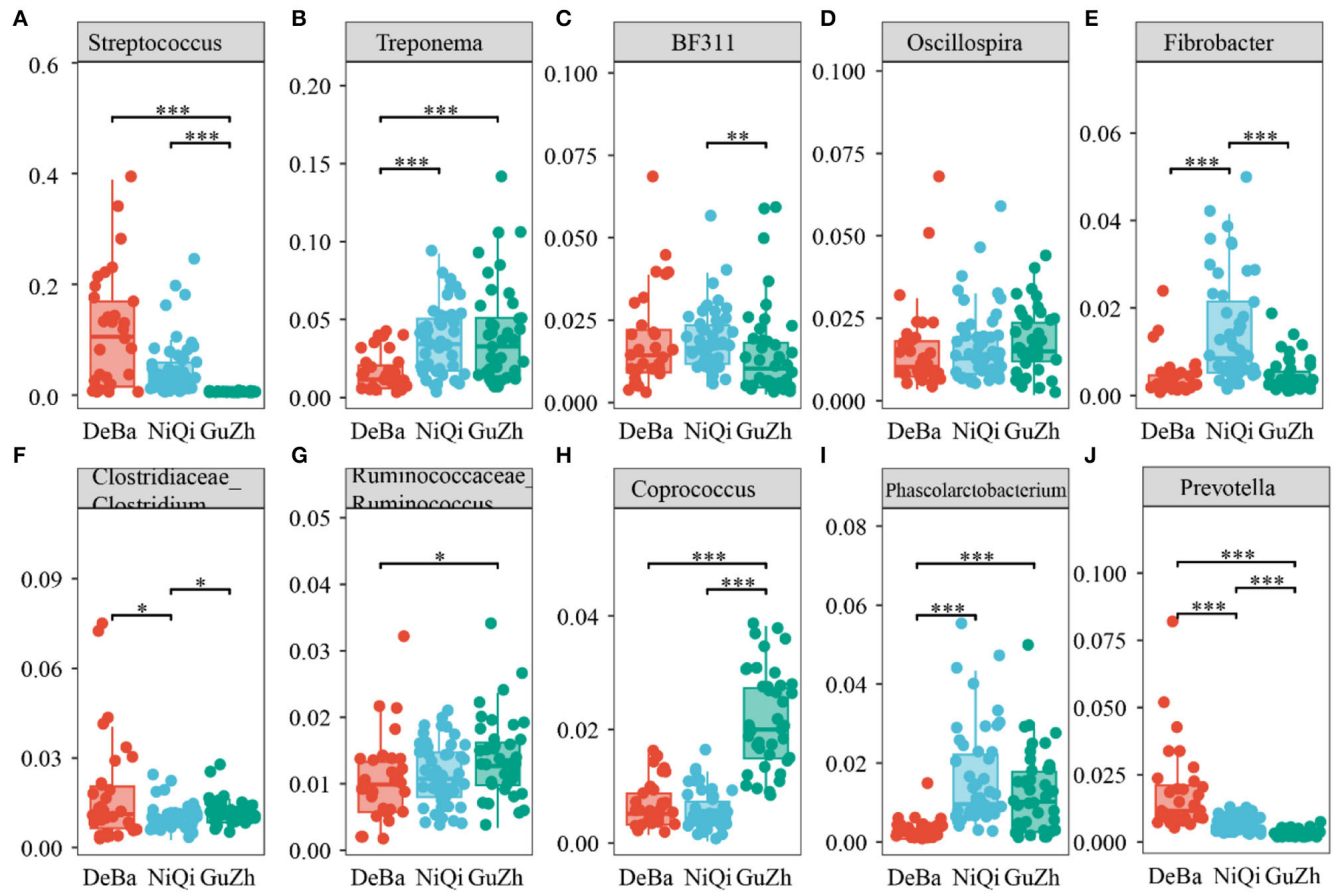
Statistical comparison of the relative abundance. **(A–J)** Comparison of dominant genera in the DeBa, NiQi, and GuZh groups (***P < 0.001, **P < 0.01, *P < 0.05).

We annotate a total of 24 phyla and 368 genera (Supplementary Figure S3). Analysis of the intestinal microbial composition of three species found that the abundance of Firmicutes was the highest, accounting for 28.87–84.98%. The second most abundant phylum was Bacteroidetes with 7.49–66.71% (Figure 3A). At the genus level, Treponema, Oscillospira, BF311 and Ruminococcaceae_Ruminococcus were rich in all samples (Figure 3B). However, bacterial taxa were different between the pony and horse fecal samples. At the phylum level, Firmicutes and Bacteroidetes showed a noteworthy difference in the three groups (Figures 4A–F). In addition, the ratio of F and B (Firmicutes and Bacteroidetes) in the GuZh horses (6.66 ± 1.28) was significantly higher than that in the DeBa (1.04 ± 0.55) and NiQi (1.36 ± 0.41) ponies (*P* < 0.01) (Table 1). The abundance of Streptococcus was significant higher in the DeBa (12.21%) and NiQi (4.56%) ponies than in the GuZh horses (0.06%). In addition, the abundance of Coprococcus in the GuZh horses (2.16%) than in the DeBa (0.63%) and NiQi (0.53%) ponies (Figures 5A–J). LEfSe analysis found that Phascolarctobacterium, Paludibacter, and Fibrobacter were markedly enriched in NiQi ponies. The relative abundances of Streptococcus and Prevotella were dramatically higher in DeBa ponies than in NiQi ponies and GuZh horses. The relative abundances of Treponema, Treponema Mogibacterium, Adlercreutzia and Blautia were dramatically higher in GuZh horses than in the DeBa ponies and NiQi ponies (Supplementary Figure S4).

**Table 1:** Proportions of Firmicutes and Bacteroidetes and the F/B ratios of three breeds horse.

**Group**	**Relative abundance of Firmicutes (Mean values ±SD)**	**Relative abundance of Bacteroidetes (Mean values ±SD)**	**Firmicutes/Bacteroidetes (F/B) ratios (Mean values ±SD)**
DeBa	48.74 ± 10.21%	46.93 ± 10.95%	1.038 ± 0.55
NiQi	51.60 ± 6.92%	39.83 ± 7.29%	1.364 ± 0.41
GuZh	77.59 ± 4.06%	11.65 ± 3.17%	6.658 ± 1.28^**^

** *P* < 0.01.

### Microflora function prediction and correlation with horse height

**Figure 6:**
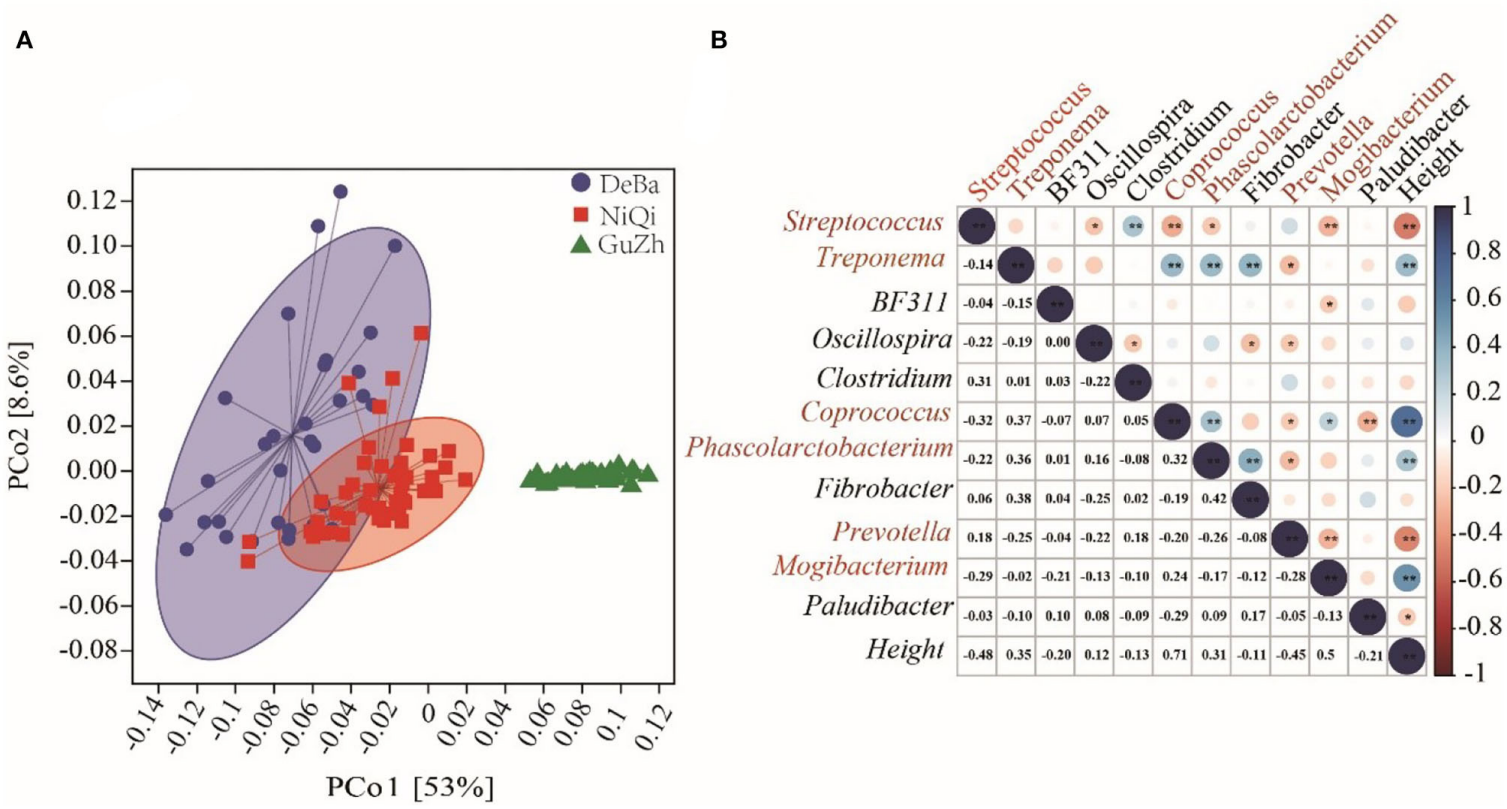
Microflora function prediction and correlation with horse height. **(A)** PCoA based on the Bray–Curtis distance of the KEGG modules in DeBa ponies, NiQi ponies, and GuZh horses. **(B)** Correlation between different bacterial genera and horse height. The size of the circle represents the correlation; the larger the circle, the stronger the correlation. The blue color denotes a positive correlation, and the red color indicates a negative correlation (**P < 0.01, *P < 0.05).

The intestinal microbial function of the three horses breeds was predicted using PICRUSt2. PCoA based on the KEGG module revealed differences in microbial function among the DeBa ponies, NiQi ponies, and GuZh horses (Figure 6A). Analogous to the results of PCoA used for assessing beta diversity, the DeBa and NiQi ponies had a similar microbial composition and parallel functions, which were quite different from those of the GuZh horse.

We selected the bacteria with relative abundance more than 1% to analyses their correlation with body height and found that six genera were significantly correlated with body height, including Streptococcus (*r* = −0.48, *P* < 0.01), Treponema (*r* = 0.35, *P* < 0.01), Coprococcus (*r* = 0.71, *P* < 0.01), Phascolarctobacterium (*r* = 0.31, *P* < 0.01), Prevotella (*r* = −0.45, *P* < 0.01) and Mogibacterium (*r* = 0.53, *P* < 0.01). Among the six genera, Coprococcus, Streptococcus, Phascolarctobacterium and Mogibacterium were classified as Firmicutes; Prevotella was classified as Bacteroidetes and Treponema was classified as Spirochaetes. We speculated that Streptococcus, Treponema, Coprococcus, Phascolarctobacterium, Prevotella, and Mogibacterium were the potential microbiota that may affected the body height (Figure 6B).

## Discussion

The association of gut microbiota diversity and function with horse health and phenotypes is currently an active area of research. In the present study, 118 equine gut microbiotas were explored through 16SrRNA high-throughput sequencing, and announced the species composition of microbes existed in the gut tract of ponies. Meanwhile we assess microbiota correlation with the height of equines. We concluded that the DeBa ponies, NiQi ponies and GuZh horses had highly diverse microbial communities. We found that the Firmicutes, Bacteroidetes, and Spirochaetes were the major bacteria phylum, which is consistent with the results of previous studies on herbivorous animals (34, 35).

However, we observed that the composition of intestinal microorganisms in the DeBa ponies and NiQi ponies was different. At the phylum level, Firmicutes is the most abundant phylum in GuZh horses, while Bacteroidetes was the most abundant phylum in DeBa and NiQi ponies. At the genus level, Streptococcus was the most abundant genus in DeBa and NiQi ponies, and Coprococcus was the most abundant genus in GuZh horses. There were many reasons for the difference among the three varieties, including differences in body size (tall vs. short), geographical distribution differences (north vs. south), and daily management (house feeding vs. grazing).

Notably, the ratio of Firmicutes and Bacteroidetes (F/B) and the relative abundance of Firmicutes in the GuZh horses were significantly higher than those in the of DeBa and NiQi ponies. Prevenient studies have shown that the higher the F/B ratio in the intestine, the stronger the ability of the host to absorb energy from food (36). Firmicutes can promote the decomposition of fibred into short-chain fatty acids (37). SCFAs (Short-chain fatty acids) can promote the absorption of calcium and induce the production of hormone-like insulin growth factor (IGF-1), which can promote bone development and affect bone health (38). A high F/B ratio and superior abundance of Firmicutes promote enhanced nutrient absorption in the GuZh horses, thus may be contributing to their big size.

Further correlation analysis suggested that Coprococcus, Mogibacterium, Treponema and Phascolarctobacterium were positively correlated with body height, whereas Streptococcus and Prevotella were negatively correlated with body height. Coprococcus is a short-chain fatty acid-producing bacterium that produces butyric acid through the phosphate transferase, the butyric acid kinase pathway and the butyryl-CoA transferase pathway. Butyric acid can promote the proliferation and development of intestinal epithelial cells. In addition, Coprococcus can use lactic acid as a substrate to produce propionic acid through the acrylic acid pathway, which is mainly involved in glycogen synthesis (39, 40). Furthermore, Treponema and Coprococcus are closely associated with pectin degradation in roughage, promote protein synthesis, and improve animal production performance (41). Phascolarctobacterium can use other bacteria to degrade succinate produced by crude fibers, and succinate can be used as a carbon source to produce SCFAs to provide nutrition for the body (42). Mogibacteriumin is associated with ammonia assimilation (43). Streptococcus mutans can stimulate the release of pro-inflammatory cytokines and promote immune regulation (44). Prevotella can degrade and utilize plant non-fibers polysaccharides such as pectin, starch and xylan (45). This means that GuZh horse may make more full use of forage, which is beneficial to its own development.

## Conclusions

The microflora analysis of equine showed that there was a significant difference in microbial composition between pony and horse. For the first time, our study characterized the Chinese ponies gut microbiota by 16S rRNA amplicon sequencing. The comparison of intestinal microbial diversity of different breeds showed that the microbial diversity of NiQi ponies was higher than that of GuZh horses. Based on clustering and PCoA analysis found that the gut microbiota of DeBa and NiQi ponies were clustered closer than those of GuZh horse. LEfSe analysis found that the content of fiber decomposing bacteria was more abundant in the gut of GuZh horses. Meanwhile, correlation analysis found that six genera were significantly correlated with equine's body height. These bacteria can degrade polysaccharides to produce SCFAs, which may affect body height. In conclusion, there may be an association between horse body height and gut microbiota. Our results can provide theoretical reference for improve health and welfare, performance, value and longevity of the horse.

## Data availability statement

The datasets generated and/or analyzed during the current study are available in the NCBI repository (https://www.ncbi.nlm.nih.gov/bioproject/, project number: PRJNA761841) with accession numbers SRR18750362 to SRR18750479.

## Ethics statement

All animal work was conducted according to the guidelines for the care and use of experimental animals established by the Ministry of Agriculture of China. And all methods are reported in accordance with ARRIVE guidelines (https://arriveguidelines.org) for the reporting of animal experiments. The project was also approved by Animal Care and Use Committee (ACUC) in Institute of Animal Sciences, Chinese Academy of Agricultural Sciences (ethical permit: IAS2019-24).

## Author contributions

SL and YZ performed data analysis and wrote the manuscript. SL, YZ, ZZ, YP, and SM conducted an animal trial and laboratory studies. YM, WL, LJ, XL, and LL conceived animal trial and revised the manuscript. All authors have read and agreed to the publication of this manuscript.

## References

[1] DeNotta SL Divers TJ. Clinical pathology in the adult sick horse: the gastrointestinal system and liver. Vet Clin North Am Equine Pract. (2020) 36:105–20. 10.1016/j.cveq.2019.11.004 31982231 PMC7127838

[2] Fan Y Pedersen O. Gut microbiota in human metabolic health and disease. Nat Rev Microbiol. (2021) 19:55–71. 10.1038/s41579-020-0433-9 32887946

[3] Underhill DM Iliev ID. The mycobiota: interactions between commensal fungi and the host immune system. Nat Rev Immunol. (2014) 14:405–16. 10.1038/nri3684 24854590 PMC4332855

[4] Qin J Li R Raes J Arumugam M Burgdorf KS Manichanh C. A human gut microbial gene catalogue established by metagenomic sequencing. Nature. (2010) 464:59–65. 10.1038/nature08821 20203603 PMC3779803

[5] Wang J Thingholm LB Skieceviciene J Rausch P Kummen M Hov JR. Genome-wide association analysis identifies variation in vitamin d receptor and other host factors influencing the gut microbiota. Nat Genet. (2016) 48:1396–406. 10.1038/ng.3695 27723756 PMC5626933

[6] Structure, function and diversity of the healthy human microbiome. Nature. (2012) 486:207–14. 10.1038/nature11234 22699609 PMC3564958

[7] Li Z Wang X Zhang Y Yu Z Zhang T Dai X. Genomic insights into the phylogeny and biomass-degrading enzymes of rumen ciliates. ISME J. (2022) 16:2775–87. 10.1038/s41396-022-01306-8 35986094 PMC9666518

[8] Song C Wang B Tan J Zhu L Lou D Cen X. Comparative analysis of the gut microbiota of black bears in china using high-throughput sequencing. Mol Genet Genomics. (2017) 292:407–14. 10.1007/s00438-016-1282-0 28028611

[9] Krautkramer KA Fan J Backhed F. Gut microbial metabolites as multi-kingdom intermediates. Nat Rev Microbiol. (2021) 19:77–94. 10.1038/s41579-020-0438-4 32968241

[10] Clark A Sallé G Ballan V Reigner F Meynadier A Cortet J. Strongyle infection and gut microbiota: profiling of resistant and susceptible horses over a grazing season. Front Physiol. (2018) 9:272. 10.3389/fphys.2018.00272 29618989 PMC5871743

[11] Destrez A Grimm P Julliand V. Dietary-induced modulation of the hindgut microbiota is related to behavioral responses during stressful events in horses. Physiol Behav. (2019) 202:94–100. 10.1016/j.physbeh.2019.02.003 30726719

[12] Zhao Y Li B Bai D Huang J Shiraigo W Yang L. Comparison of fecal microbiota of Mongolian and thoroughbred horses by high-throughput sequencing of the v4 region of the 16s rRNA gene. Asian-Australas J Anim Sci. (2016) 29:1345–52. 10.5713/ajas.15.0587 26954132 PMC5003997

[13] Metcalf JL Song SJ Morton JT Weiss S Seguin-Orlando A Joly F. Evaluating the impact of domestication and captivity on the horse gut microbiome. Sci Rep. (2017) 7:15497. 10.1038/s41598-017-15375-9 29138485 PMC5686199

[14] Massacci FR Clark A Ruet A Lansade L Costa M Mach N. Inter-breed diversity and temporal dynamics of the fecal microbiota in healthy horses. J Anim Breed Genet. (2020) 137:103–20. 10.1111/jbg.12441 31523867

[15] Ang L Vinderola G Endo A Kantanen J Jingfeng C Binetti A. Gut microbiome characteristics in feral and domesticated horses from different geographic locations. Commun Biol. (2022) 5:172. 10.1038/s42003-022-03116-2 35217713 PMC8881449

[16] Aricha H Simujide H Wang C Zhang J Lv W Jimisi X. Comparative analysis of fecal microbiota of grazing mongolian cattle from different regions in inner Mongolia, china. Animals. (2021) 11:1938. 10.3390/ani11071938 34209653 PMC8300212

[17] Chen C Zhou Y Fu H Xiong X Fang S Jiang H. Expanded catalog of microbial genes and metagenome-assembled genomes from the pig gut microbiome. Nat Commun. (2021) 12:1106. 10.1038/s41467-021-21295-0 33597514 PMC7889623

[18] Levin D Raab N Pinto Y Rothschild D Zanir G Godneva A. Diversity and functional landscapes in the microbiota of animals in the wild. Science. (2021) 372:5352. 10.1126/science.abb5352 33766942

[19] Ma Y Ma S Chang L Wang H Ga Q Ma L. Gut microbiota adaptation to high altitude in indigenous animals. Biochem Biophys Res Commun. (2019) 516:120–6. 10.1016/j.bbrc.2019.05.085 31196622

[20] Yang H Xiu W Liu J Yang Y Zhang Y Zheng Y. Characteristics of the intestinal microorganisms in middle-aged and elderly patients: effects of smoking. Acs Omega. (2022) 7:1628–38. 10.1021/acsomega.1c02120 35071858 PMC8771693

[21] Chen M Fan HN Chen XY Yi YC Zhang J Zhu JS. Alterations in the saliva microbiome in patients with gastritis and small bowel inflammation. Microb Pathog. (2022) 165:105491. 10.1016/j.micpath.2022.105491 35318071

[22] Bokulich NA Kaehler BD Rideout JR Dillon M Bolyen E Knight R. Optimizing taxonomic classification of marker-gene amplicon sequences with qiime 2's q2-feature-classifier plugin. Microbiome. (2018) 6:90. 10.1186/s40168-018-0470-z 29773078 PMC5956843

[23] Callahan BJ McMurdie PJ Rosen MJ Han AW Johnson AJ Holmes SP. Dada2: high-resolution sample inference from illumina amplicon data. Nat Methods. (2016) 13:581–3. 10.1038/nmeth.3869 27214047 PMC4927377

[24] Bolyen E Rideout JR Dillon MR Bokulich NA Abnet CC Al-Ghalith GA. Reproducible, interactive, scalable and extensible microbiome data science using qiime 2. Nat Biotechnol. (2019) 37:852–7. 10.1038/s41587-019-0209-9 31341288 PMC7015180

[25] Koljalg U Nilsson RH Abarenkov K Tedersoo L Taylor AF Bahram M. Towards a unified paradigm for sequence-based identification of fungi. Mol Ecol. (2013) 22:5271–7. 10.1111/mec.12481 24112409

[26] Liu Y Bailey KE Dyall-Smith M Marenda MS Hardefeldt LY Browning GF. Faecal microbiota and antimicrobial resistance gene profiles of healthy foals. Equine Vet J. (2021) 53:806–16. 10.1111/evj.13366 33030244

[27] Amato KR Yeoman CJ Kent A Righini N Carbonero F Estrada A. Habitat degradation impacts black howler monkey (*alouatta pigra*) gastrointestinal microbiomes. ISME J. (2013) 7:1344–53. 10.1038/ismej.2013.16 23486247 PMC3695285

[28] Fan L Wang Z Chen M Qu Y Li J Zhou A. Microbiota comparison of pacific white shrimp intestine and sediment at freshwater and marine cultured environment. Sci Total Environ. (2019) 657:1194–204. 10.1016/j.scitotenv.2018.12.069 30677886

[29] Hong M Peng G Keyhani NO Xia Y. Application of the entomogenous fungus, metarhizium anisopliae, for leafroller (*cnaphalocrocis medinalis*) control and its effect on rice phyllosphere microbial diversity. Appl Microbiol Biotechnol. (2017) 101:6793–807. 10.1007/s00253-017-8390-6 28695229

[30] Schloss PD Gevers D Westcott SL. Reducing the effects of pcr amplification and sequencing artifacts on 16s rRNA-based studies. PLoS One. (2011) 6:e27310. 10.1371/journal.pone.0027310 22194782 PMC3237409

[31] Jiang X Peng X Deng G Sheng H Wang Y Zhou H. Illumina sequencing of 16s rrna tag revealed spatial variations of bacterial communities in a mangrove wetland. Microb Ecol. (2013) 66:96–104. 10.1007/s00248-013-0238-8 23649297

[32] Segata N Izard J Waldron L Gevers D Miropolsky L Garrett WS. Metagenomic biomarker discovery and explanation. Genome Biol. (2011) 12:R60. 10.1186/gb-2011-12-6-r60 21702898 PMC3218848

[33] Bhandari SK Nyachoti CM Krause DO. Raw potato starch in weaned pig diets and its influence on post-weaning scours and the molecular microbial ecology of the digestive tract. J Anim Sci. (2009) 87:984–93. 10.2527/jas.2007-0747 18952739

[34] Zhang H Shao M Huang H Wang S Ma L Wang H. The dynamic distribution of small-tail han sheep microbiota across different intestinal segments. Front Microbiol. (2018) 9:32. 10.3389/fmicb.2018.00032 29445360 PMC5797768

[35] Indugu N Vecchiarelli B Baker LD Ferguson JD Vanamala J Pitta DW. Comparison of rumen bacterial communities in dairy herds of different production. BMC Microbiol. (2017) 17:190. 10.1186/s12866-017-1098-z 28854878 PMC5577838

[36] Ley RE Backhed F Turnbaugh P Lozupone CA Knight RD Gordon JI. Obesity alters gut microbial ecology. Proc Natl Acad Sci USA. (2005) 102:11070–5. 10.1073/pnas.0504978102 16033867 PMC1176910

[37] Jami E Israel A Kotser A Mizrahi I. Exploring the bovine rumen bacterial community from birth to adulthood. ISME J. (2013) 7:1069–79. 10.1038/ismej.2013.2 23426008 PMC3660679

[38] Yuan L Qi A Cheng Y Sagen G Qu Y Liu B. Fecal microbiota of three bactrian camels (*camelus ferus* and *camelus bactrianus*) in china by high throughput sequencing of the v3-v4 region of the 16s rRNA gene. J Arid Land. (2017) 9:153–9. 10.1007/s40333-016-0026-7

[39] Bultman SJ. Molecular pathways: gene-environment interactions regulating dietary fiber induction of proliferation and apoptosis via butyrate for cancer prevention. Clin Cancer Res. (2014) 20:799–803. 10.1158/1078-0432.CCR-13-2483 24270685 PMC3944646

[40] Louis P Hold GL Flint HJ. The gut microbiota, bacterial metabolites and colorectal cancer. Nat Rev Microbiol. (2014) 12:661–72. 10.1038/nrmicro3344 25198138

[41] Liu J Wang JK Zhu W Pu YY Guan LL Liu JX. Monitoring the rumen pectinolytic bacteria treponema saccharophilum using real-time pcr. Fems Microbiol Ecol. (2014) 87:576–85. 10.1111/1574-6941.12246 24289046

[42] Morotomi M Nagai F Sakon H Tanaka R. Paraprevotella clara gen. Nov, Sp Nov And paraprevotella xylaniphila sp Nov, Members of the family ‘prevotellaceae' isolated from human faeces. Int J Syst Evol Microbiol. (2009) 59:1895–900. 10.1099/ijs.0.008169-0 19567577

[43] Gomes CAK Granja-Salcedo YT Messana JD Carneiro DSV Generoso GM Detogni CP. Rumen bacterial diversity in relation to nitrogen retention in beef cattle. Anaerobe. (2021) 67:102316. 10.1016/j.anaerobe.2020.102316 33383197

[44] Zvanych R Lukenda N Li X Kim JJ Tharmarajah S Magarvey NA. Systems biosynthesis of secondary metabolic pathways within the oral human microbiome member streptococcus mutans. Mol Biosyst. (2015) 11:97–104. 10.1039/c4mb00406j 25209237

[45] Mohammadzadeh H Yanez-Ruiz DR Martinez-Fernandez G Abecia L. Molecular comparative assessment of the microbial ecosystem in rumen and faces of goats fed alfalfa hay alone or combined with oats. Anaerobe. (2014) 29:52–8. 10.1016/j.anaerobe.2013.11.012 24333680

