# Supplementary material for: Diversity of the fecal microbiota in Chinese ponies
Source: Front Vet Sci. 2023 Jan 26;10:1102186. doi: 10.3389/fvets.2023.1102186 (PMC9909481; doi:10.3389/fvets.2023.1102186)
Supplement: Supplementary file 1 [file Data_Sheet_1.docx]

**Supplementary materials:**

Diversity of the faecal microbiota in Chinese ponies

Shipeng Lv^123#^, Yanli Zhang^23#^, Zhengkai Zhang^23^, Sihan Meng^23^, Yabin Pu^23^, Xuexue Liu^234^, Lingling Liu^1^, Yuehui Ma^23^, Wujun Liu^1*^, Lin Jiang^23*^

#These authors contributed equally to this article.

*Correspondence:

Corresponding Author

E-mail: lwj_ws@163.com (Wujun Liu)

E-mail: jianglin@caas.cn (Lin Jiang)

^1^College of Animal Science, Xinjiang Agricultural University, Urumqi 830052, China.

^2^Laboratory of Animal (Poultry) Genetics Breeding and Reproduction, Ministry of Agriculture, Institute of Animal Science, Chinese Academy of Agricultural Sciences (CAAS), Beijing 100193, P. R. China.

^3^CAAS-ILRI Joint Laboratory on Livestock and Forage Genetic Resources, Institute of Animal Science, Chinese Academy of Agricultural Sciences (CAAS), Beijing, 100193, P.R. China.

^4^Centre d’Anthropobiologie et de Génomique de Toulouse, Université Paul Sabatier, 37 allées Jules Guesde, 31000 Toulouse, France

Table S1. Information on the equines used in this study

| **Breed** | **Abbr** | **Distribution** | **Diet** | **Gender** | **Sampling Size** | **Age** | **Height(cm)** |
| --- | --- | --- | --- | --- | --- | --- | --- |
| Debao pony | DeBa | Debao County in Guangxi | Alfalfa, Concentrate supplement | male | 13 | 9.07±5.48 | 99.68±7.75 |
|  |  |  |  | female | 18 | 10.15±5.38 | 101.24±5.67 |
| Ningqiang pony | NiQi | Ningqiang County in Shannxi | Pennisetum purpureumcv.Guimu-1, Concentrate supplement | male | 20 | 4.89±2.81 | 108.94±6.73 |
|  |  |  |  | female | 27 | 7±2.95 | 113.92±2.74 |
| Guanzhong horse | GuZh | Baoji County in Shannxi | ensilage, Concentrate supplement | male | 6 | 5.50±2.42 | 158.16±1.67 |
|  |  |  |  | female | 34 | 4.28±2.89 | 150.38±4.71 |


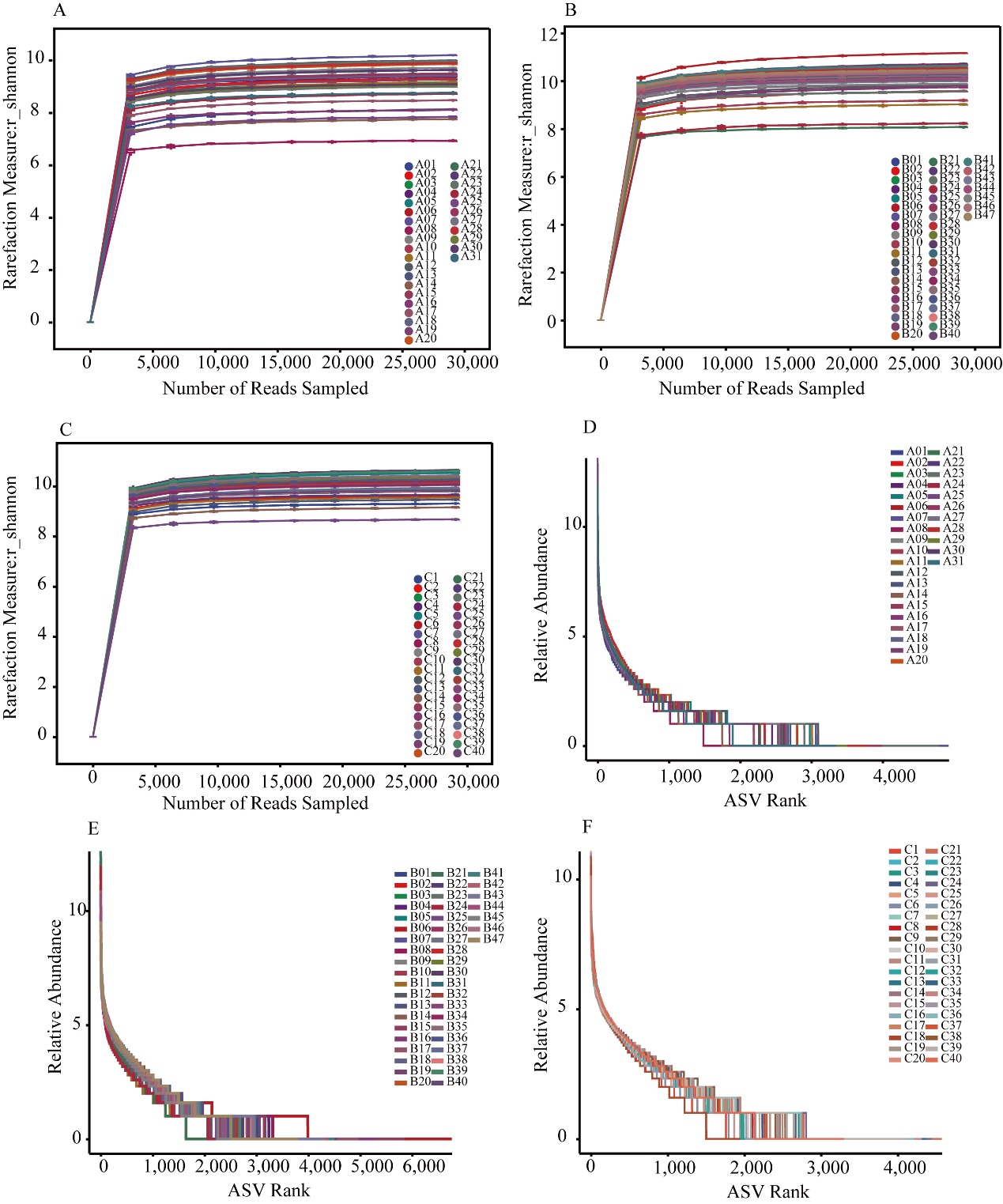
Fig S1. A-C is shannon-Wiener curves of 31 samples from DeBa pony(A), 47 samples from NiQi pony(B), and 40 samples from GuZh horse(C); D-F is ASV Rank-Abundance curves of 31 samples from DeBa pony(D), 47 samples from NiQi pony(E), and 40 samples from GuZh horse(F).


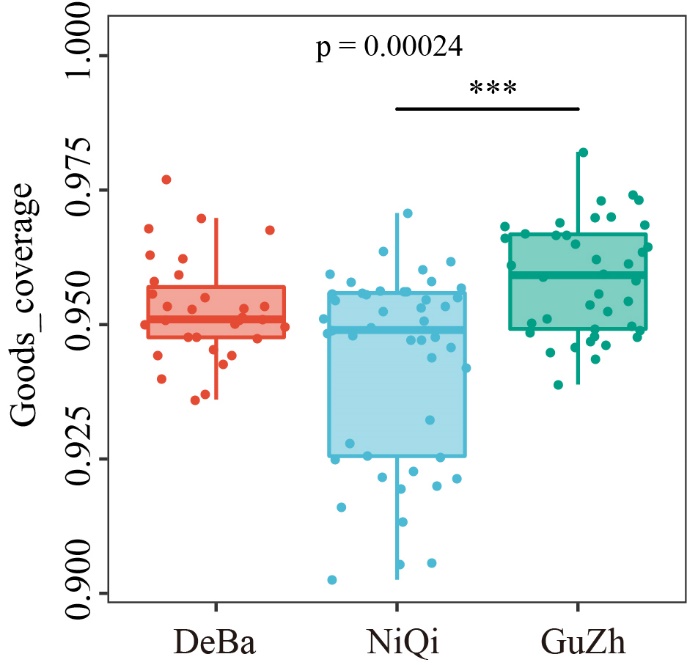


Fig S2 The Good’s coverage of DeBa ponies, NiQi ponies, and GuZh horses. Statistical method: one-way ANOVA with Tukey’s post-hoc test. (*** P<0.001, ** P < 0.01, * P < 0.05)

**
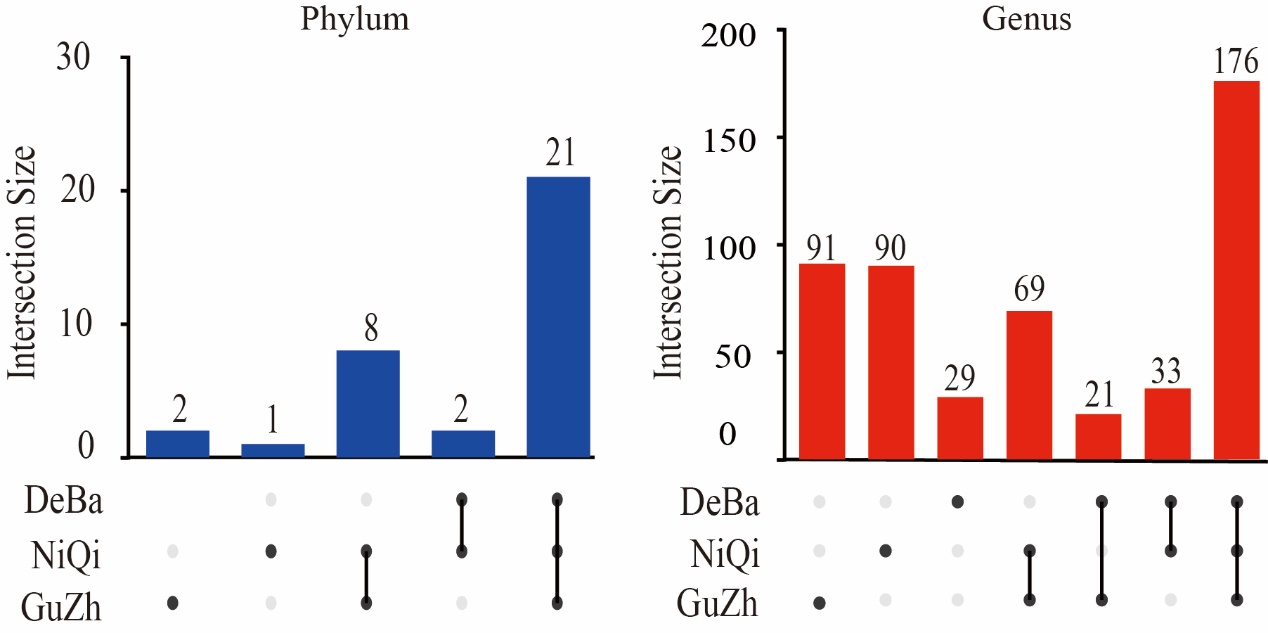
Fig S3** Upset diagrams at the classification level of phylum and genus. Each point in the node matrix on the lower right represents the unique phylum (genus) the three horse breeds, the connection between the nodes represents the common phylum (genus) of the statistically connected set, and the column on the upper right represents the phylum owned by the set corresponding to the connection of the lower node (the histogram of the number of genera). The left graph is the number of annotated to phyla, and the right is the number of annotated to genera.


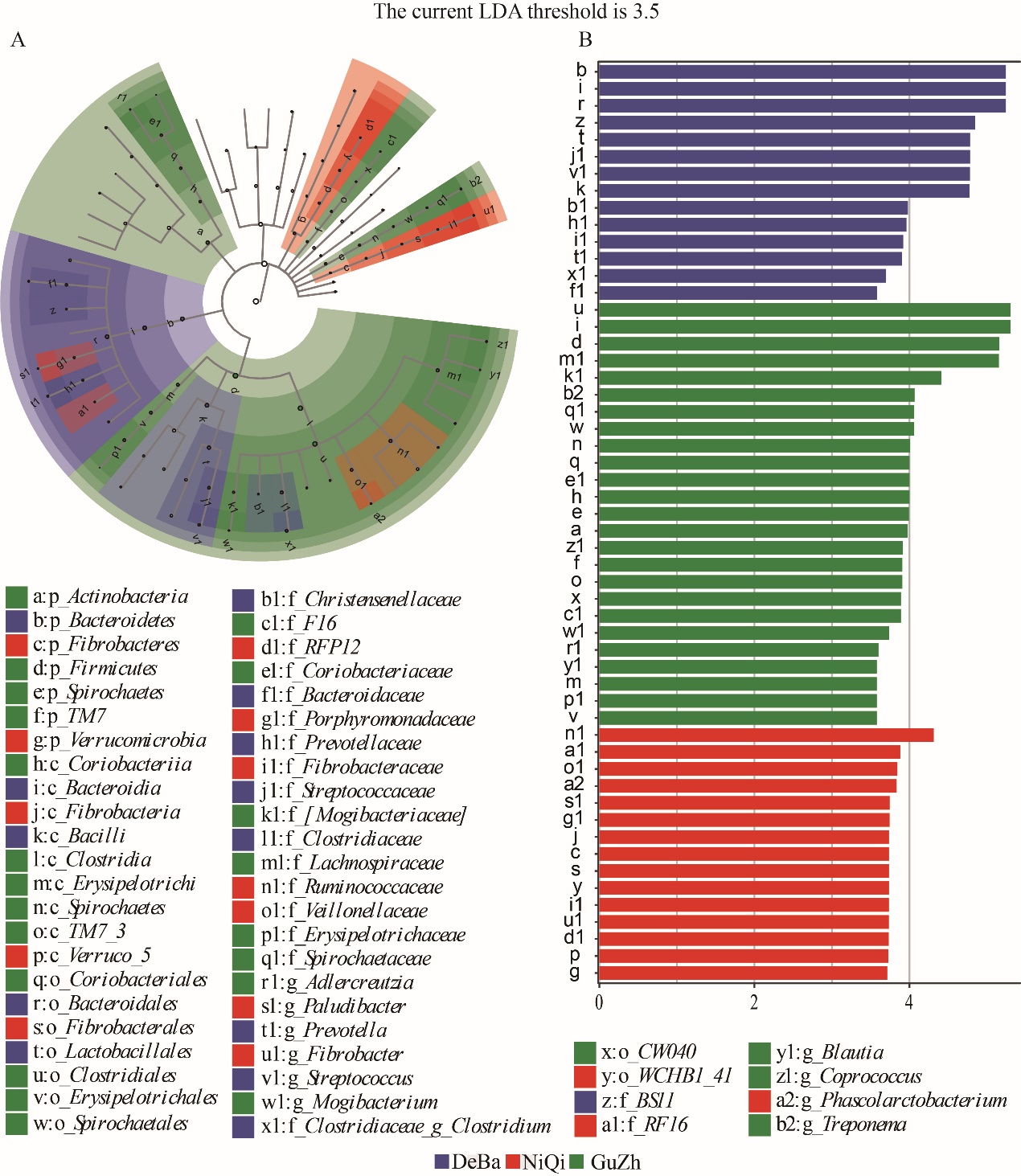


**Fig. S4** Bacterial taxa that differed significantly among the DeBa ponies, NiQi ponies and GuZh horses were identified via linear discriminant analysis (LDA>3.5) effect size (LEfSe) using default parameters.
